# Effects of non-pharmacological interventions on ulcer healing in patients with diabetic foot: a network meta-analysis of randomized controlled trials

**DOI:** 10.3389/fendo.2026.1811595

**Published:** 2026-03-26

**Authors:** Jiaxing Zheng, Danna Xie, Minyu Wu, Hongtao Xu, Siyin Lin, Lili Dong

**Affiliations:** 1 The Second Affiliated Hospital of Shantou University Medical College, Shantou, China; 2 Shantou University Medical College, Shantou, China; 3 Shantou University Medical College Cancer Hospital, Shantou, China

**Keywords:** diabetic foot ulcer, healing rate, healing time, network meta-analysis, non-pharmacological intervention, nursing, wound care

## Abstract

**Background:**

Diabetic Foot Ulcers are a serious complication of diabetes, and the clinical treatment is challenging. In this study, network meta-analysis was used to evaluate the effect of different non-drug interventions on Diabetic Foot Ulcer healing and provide evidence to support clinical decision-making.

**Methods:**

Data sources: 8 databases, including PubMed, Embase, Cochrane, WOS, CNKI, Wanfang Data, CBM, and VIP, were systematically searched. Literature screening: According to the principle of PICOS, two researchers independently screened. Quality evaluation: Two researchers independently used the Cochrane bias risk assessment tool (RoB 2.0) to evaluate the risk of bias. Statistical analysis: by NMA of the random effects model under the framework of frequency.

**Results:**

A total of 24 randomized controlled trials involving 15 types of interventions were included. The overall quality of the included literature was moderate. The results of NMA showed that Negative Pressure Wound Therapy + Standard Care had the best effect on 12-week healing rate. Compared with Standard Care, Gas Therapy+ Dressing Therapy + Standard Care had the best healing time.

**Conclusion:**

Negative Pressure Wound Therapy + Standard Care may be more beneficial for improving the 12-week healing rate of Diabetic Foot Ulcer, while Gas Therapy + Dressing Therapy + Standard Care may show potential advantages in shortening healing time. However, these results rely on a limited number of small-sample studies with wide confidence intervals, indicating limited certainty of evidence. Consequently, the SUCRA rankings should be interpreted with caution as they are primarily exploratory; further high-quality RCTs are required to confirm these findings.

**Systematic Review Registration:**

https://www.crd.york.ac.uk/prospero/, identifier CRD420251122143.

## Introduction

Diabetes is one of the leading causes of death and disability worldwide (1). The research published in *The Lancet* in 2023 pointed out that (1), as of 2021, the number of adults with diabetes worldwide reached 529 million people, and it is expected that by 2050, the world will have more than 1.31 billion people with diabetes. This data underscores the importance of diabetes and its complications management. As one of the most serious chronic complications of diabetes, Diabetic Foot Ulcer (DFU) has become a major challenge in the field of global public health (2, 3). DFU occurs in 19% to 34% of diabetic patients worldwide for the rest of their lives (4, 5). DFU is easy to relapse. The recurrence rate is about 40% within 1 year after healing and up to 65% within 5 years (6). About 50% to 60% of DFU are infected, and 20% of moderate to severe infections require amputation (7), and the 5-year mortality rate in patients with DFU is as high as 50-70%, much higher than in patients without DFU (4–7). DFU significantly increases the consumption of medical resources and economic burden. The number of hospitalization days, emergency and outpatient visits of DFU patients were significantly higher than those of patients without DFU, and the average annual medical expenses increased by $ 10,000 to $ 30,000 (8). In the United States, the annual direct medical costs associated with DFU are as high as $ 6 billion, accounting for about 10% of the total cost of diabetic complications (9). In developing countries, patients and families often fall into catastrophic expenditure due to DFU, and indirect losses (such as disability, unemployment) account for more than 70% of the total burden (10). In view of the high morbidity, high disability, high mortality, and heavy economic burden of DFU, it is particularly urgent to find effective treatment methods.

At present, the clinical treatment of DFU is based on Standard Care (SC), including debridement, dressing change, foot decompression, peripheral vascular assessment, infection control, and refined management of blood glucose (11, 12). High-quality studies and the latest meta-analysis have confirmed that the complete healing rate of DFU patients receiving 12 weeks of SC was only 24% -33%, indicating that the traditional regimen had limited efficacy and was far from meeting the clinical needs for efficacy (13, 14).

Due to the insufficient efficacy of SC, there have been many more effective interventions in recent years, and the number of related RCTs has increased significantly (15–45), including Negative Pressure Wound Therapy (NPWT) (33), Gas Therapy (GT) (46), Phototherapy (P) (41), Dressing Therapy (DT) (38), and so on. For the evaluation of the efficacy of such new non-drug interventions, some studies have used traditional meta-analysis methods to compare the efficacy of single interventions (such as NPWT *vs* SC) (47, 48). One study showed that the healing rate of the NPWT group was significantly higher than that of the standard group (OR = 3.60,95% CI: 2.38 to 5.45) (48). However, traditional Meta-analysis can only achieve direct comparison between the two interventions, and it is difficult to find out which intervention is the best for DFU among many new interventions.

At present, for the evaluation of the efficacy of different interventions for DFU, some studies have used the network meta-analysis (NMA) to conduct an integrated analysis of various interventions, trying to provide evidence-based reference for clinical decision-making (49–56). For example, one study showed that stem cell therapy was the optimal intervention in terms of DFU healing rate (OR = 5.71,95% CI: 2.64 to 12.34, SUCRA = 89.7%) (51). Meanwhile, another study showed that the optimal intervention for DFU healing rate was stem cells combined with platelet-rich plasma (OR = 22.0,95% CI: 2.5 to 23.0, SUCRA = 83.56%) (50). In addition, another study showed that the best gas intervention for DFU healing rate was hyperbaric oxygen therapy, and the MD value was -2.71 (95% CI: -4.85 to -1.34) compared with routine nursing (49).

However, there are still limitations in the clinical application of existing studies: on the one hand, the included intervention programs mostly include drug interventions such as epidermal growth factor EGF or other external drugs (52, 53). It is difficult for medical staff to extract independent evidence that is only applicable to non-drug intervention for DFU patients with drug allergy and clear refusal of drug treatment. On the other hand, at the level of outcome indicators, although some studies have conducted subgroup analysis by treatment time ‘ ≤ 6 weeks ‘ and ‘ > 6 weeks ‘ (49), most studies have not limited a unified time node, which may lead to a decrease in the reliability of the results. The UK’s National Diabetes Foot Care Audit (NDFA), an authoritative national audit project in the field of DFU, explicitly incorporates 12-week outcomes into a standardized assessment system (57, 58). In addition, at the population inclusion level, most studies did not strictly limit the disease staging of DFU patients (50, 51). If the internationally recognized standardized staging system (such as Wagner classification and TEXAS classification) is not adopted, or the population is defined by descriptive classification without quantitative criteria such as mild or severe, it may be included in Wagner Grade 0 (no ulcer only high-risk factors, no targeted wound intervention) and Wagner Grade 5 (full foot gangrene, ineffective conservative treatment requires surgical intervention) patients, thus introducing a large number of clinical heterogeneity, which may affect the reliability of evidence and clinical transformation value. In summary, there are still limitations in the existing network evidence of DFU intervention: there is no NMA that only includes non-drug intervention, a unified outcome evaluation time node, and that strictly limits the subjects according to standardized staging.

Therefore, in order to fill this gap and accurately promote the early healing of DFU, this study aims to compare the effects of different non-drug interventions through NMA. Among them, ‘ healing effect ‘ refers specifically to the 12-week ulcer healing rate and ulcer healing time in this paper, and does not involve indicators such as amputation rate, mortality rate, and treatment cost.

## Methods

This study is based on the statement of PRISMA (Preferred Reporting Items for Systematic Reviews and Meta-Analyses) (59, 60), and the Cochrane Handbook for Systematic Review of interventions. The research program has been registered in the PROSPERO international prospective system evaluation registration platform (registration number: CRD420251122143).

### Search strategy

The search scope included 8 databases: PubMed, Embase, Cochrane, WOS, CNKI, Wanfang Data, CBM, VIP. The search time limit is from the establishment of the database to August 2025, and the search strategy of combining subject words with free words is adopted. We have used NOT ‘ drug intervention ‘ and ‘ review ‘ to roughly exclude some studies that are not related to the topic. In addition, by manually reading the references and clinical intervention guidelines included in the study (61), possible eligible studies were screened according to the title, and finally, the full text was read to determine whether to include the review. Take PubMed as an example, see **Table 1** for details.

**Table 1:** The search strategy of PubMed.

Line	Search strategy
14	12 NOT 13
13	(review[Publication Type]) OR (meta[Title/Abstract])
12	11 NOT 7
11	3 AND 6 AND 10
10	8 OR 9
9	(((((((((((((Wound Healing[Title/Abstract]) OR (granulation, wound[Title/Abstract])) OR (repair, wound[Title/Abstract])) OR (wound regeneration[Title/Abstract])) OR (wound repair[Title/Abstract])) OR (Healings, Wound[Title/Abstract])) OR (Healing, Wound[Title/Abstract])) OR (Wound Healings[Title/Abstract])) OR (Wound repair[Title/Abstract])) OR (Ulcer healing[Title/Abstract])) OR (Chronic wound healing[Title/Abstract])) OR (Healing efficacy[Title/Abstract])) OR (Healing rate[Title/Abstract])) OR (Re-Epithelialization[Title/Abstract])
8	“Wound Healing”[Mesh]
7	“drug therapy” [Subheading]
6	4 OR 5
5	((((((((((((Diabetic Foot[Title/Abstract]) OR (Foot, Diabetic[Title/Abstract])) OR (Diabetic Feet[Title/Abstract])) OR (Feet, Diabetic[Title/Abstract])) OR (Foot Ulcer, Diabetic[Title/Abstract])) OR (Diabetic foot ulcer[Title/Abstract])) OR (Diabetic foot lesions[Title/Abstract])) OR (diabetic foot disease[Title/Abstract])) OR (Diabetic lower extremity ulcer[Title/Abstract])) OR (Foot problems in diabetes[Title/Abstract])) OR (Diabetic gangrene of foot[Title/Abstract])) OR (Diabetic foot infection[Title/Abstract])) OR (diabetic foot syndrome[Title/Abstract])
4	“Diabetic Foot”[Mesh]
3	1 OR 2
2	“Randomized Controlled Trials as Topic”[Mesh]
1	“Randomized Controlled Trial” [Publication Type]

### Inclusion and exclusion criteria

According to the PICOS principle, the literature screening criteria were formulated.

#### Inclusion criteria

P: The subjects should meet the WHO (1999) diagnostic criteria for diabetes, and be diagnosed as DFU by clinical examination, and clear ulcer grading (such as Wagner Grades 1-4, University of Texas Grades 1-3).

I: Non-drug single or combined intervention measures.

C: non-drug single, combined interventions, routine care, or routine care + placebo.

O: 12 weeks ulcer healing rate, if the number of reports is divided by the number of healings by the original sample size calculation, ulcer healing time must report the mean and standard deviation.

S: RCTs; no matter whether the blind method is adopted, there is no restriction on publication date or publication status.

#### Exclusion criteria

P: non-diabetic patients or non-foot ulcers; there was no indication of ulcer grade, no ulcer, only high-risk factors, no need for targeted wound intervention or full-foot gangrene, ineffective conservative treatment requiring surgical intervention, and end-stage amputation cases.

I: Intervention measures include drug therapy, such as local or systemic use of antibiotics, growth factor drugs, etc.; comparison between similar interventions, such as alginate dressings *vs* foam dressings.

C: SC has no clear operation standard, or is seriously inconsistent with clinical SC, and cannot guarantee the consistency of the control study.

O: healing rate is not 12 weeks for the node; the definition of healing is inconsistent (non-100% epithelialization of the wound); healing time reported as median form; the original data cannot extract specific quantitative results.

S: non-randomized controlled trials, non-Chinese and English language reports, dissertations.

### Literature screening

After obtaining the bibliography through retrieval, the literature management software NoteExpress (version 4.2.0.10240) is used for unified management, and its intelligent deduplication function is used to eliminate duplicate records. Then, following the PRISMA guidelines (59, 60), two nursing postgraduates who had undergone professional evidence-based learning independently screened the titles and abstracts and reviewed the full text. According to the predetermined PICOS standard, the literature of the preliminary screening is divided into (qualified/unqualified/to be discussed), and then the literature classified by the two people is compared. The inconsistent entry into the discussion determines whether the preliminary screening is qualified. Finally, the literature of the preliminary screening is downloaded. After reading the full text by two people, the same classification as the preliminary screening is carried out, and the literature of the second screening is unanimously judged to be qualified for the data extraction stage. If there are differences in all screening steps, they are resolved through discussion and consultation with another researcher’s diabetic foot care expert to ensure the accuracy of the results. The final screening process and results are fully presented through the PRISMA flow chart, and the number and reasons for the exclusion of the literature are recorded in detail.

### Data extraction

In this study, a pre-designed data extraction table was used. Two researchers independently extracted relevant data, including the first author, publication year, study area, study design, sample size, study population characteristics (gender, age, wound stage), interventions, control measures, intervention time, 12-week healing rate, and healing time.

### Quality evaluation

In this study, the Revised Randomized Trial Bias Risk Assessment Tool (RoB2) recommended by Cochrane was used to assess the risk of bias in all included studies (62). Two researchers independently judged each study as ‘ low risk ‘, ‘ with some concerns ‘, or ‘ high risk ‘ in the five core areas of randomization process, deviation from established intervention, missing outcome data, outcome measurement, and selective reporting results. The differences were resolved through consultation or discussion by a third researcher, and the overall bias risk of the study was determined according to the strictest dimension principle.

### Analytical method

All analyses were performed in Stata 17.0. The 12-week healing rate was expressed as an odds ratio (OR) and 95% confidence interval (CI), and the healing time was expressed as mean difference (MD) and 95% CI. The traditional meta-analysis was performed for all direct comparisons, and the heterogeneity between studies was tested using the Cochrane Q test (test level *P* < 0.05) and *I^2^* statistics (63). If *I^2^* ≤ 50% and the Q test *P* value > 0.05, it is considered that the heterogeneity between studies is small, and the fixed effect model is adopted. Otherwise, the random effect model is used. In cases of significant heterogeneity, subgroup analyses will be conducted based on pre-specified potential moderators, including Wagner grade of diabetic foot ulcers, intervention type, treatment cycle, and country of origin, to explore and explain the sources of heterogeneity. However, considering the various types of interventions that may be included in this study (such as DT, GT, etc.) (61), there may be significant differences in the mechanism of action and efficacy, which may be an important reason for the overall heterogeneity. Therefore, we first perform traditional meta-analysis on all direct comparisons to obtain the combined effect size, then classify different interventions, and perform heterogeneity tests on comparisons containing ≥ 2 studies to provide a basis for subsequent NMA.

This NMA is based on the frequency framework (64). It is proposed to construct a network diagram, and the classified interventions are set as nodes in the network, and the size of the nodes is determined by the sample size; the connection between nodes represents the direct comparison relationship between different interventions, and the thickness of the connection matches the frequency of such direct comparison studies. The evaluation of the transitivity hypothesis needs to be completed by analyzing the characteristics of the research, and combined with the inspection of the existing complete evidence network, the indirect evidence is comprehensively considered (65).

In order to test the significant difference between the direct comparison and the indirect comparison of each intervention measure, the Wald test was used to evaluate the global inconsistency, and the ring inconsistency test and the node splitting method were used to evaluate the local inconsistency (66). P value > 0.05 indicated that direct evidence was consistent with indirect evidence. In addition, Surface Under the Cumulative Ranking Curve (SUCRA) is used (67). Quantitative evaluation of the probability-based relative efficacy ranking of various non-drug interventions on the 12-week healing rate and healing time of DFU. Visual correction was used to compare funnel plots, and their symmetry was visually examined. Egger’s test and Begg’s test (*α* = 0.05) were used for further verification to assess publication bias (68). Finally, through the systematic exclusion of individual studies and then re-meta-analysis, sensitivity analysis was performed to test the robustness of the results. Furthermore, the Grading of Recommendations Assessment, Development and Evaluation (GRADE) approach was used to assess the certainty of evidence for key comparisons and outcomes.

## Result

### Literature screening

**Figure 1:**
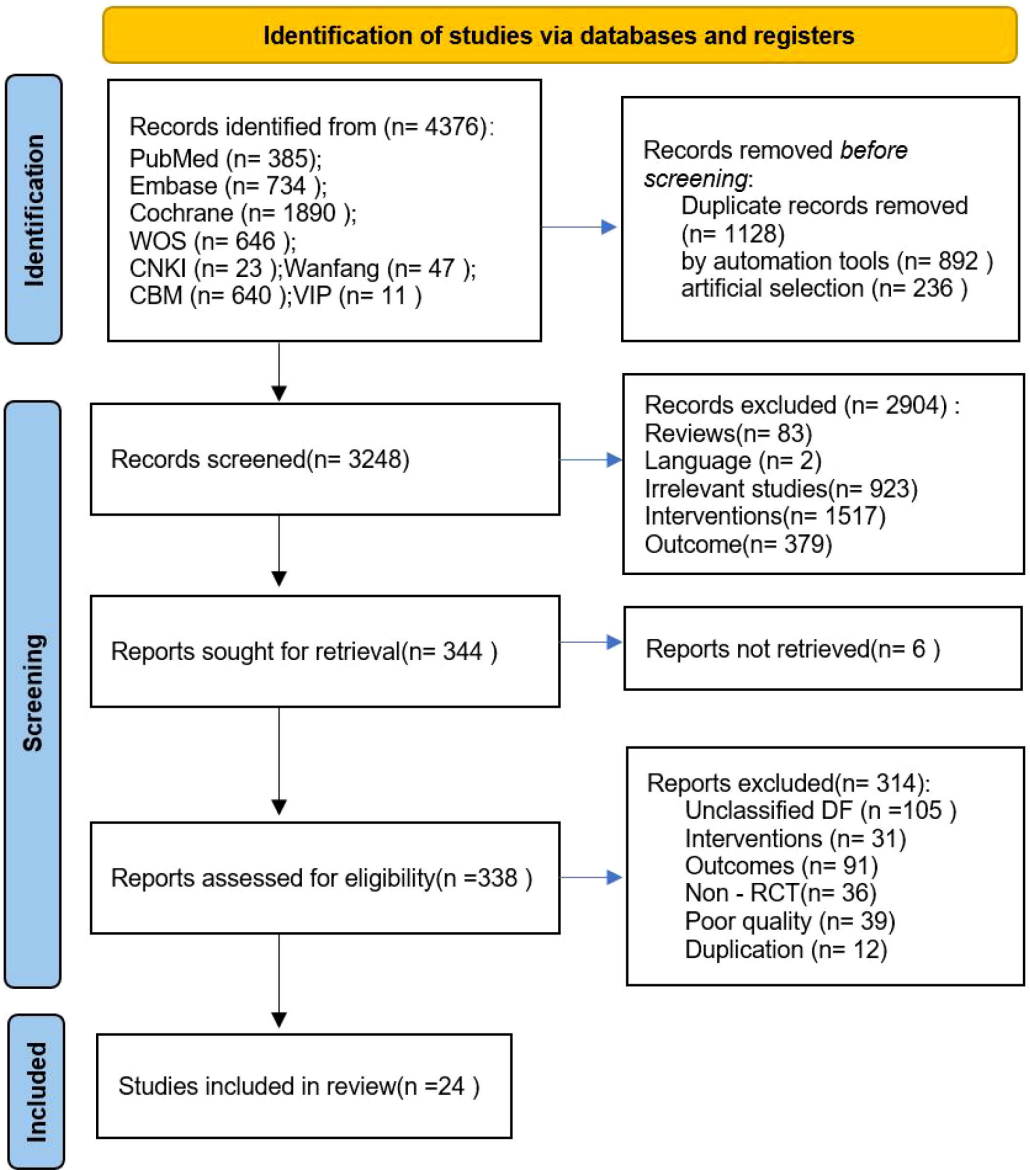
Flow diagram for the search and selection of the included studies. WOS, Web of Science; CNKI, China National Knowledge Infrastructure; CBM, Chinese Biomedicine Literature.

The screening process of this study strictly followed the PRISMA specification, and the results are shown in **Figure 1**. Through the systematic search of PubMed, Embase, Cochrane, WOS, CNKI, Wanfang Data, CBM, and VIP databases, 4376 related articles were initially identified. After removing 1128 duplicate articles, the titles and abstracts of the remaining 3248 articles were screened. According to the preset inclusion and exclusion criteria, 2904 articles, such as inconsistent research types and mismatched intervention measures, were excluded. A total of 344 articles were evaluated, and 314 were excluded due to unclear ulcer grading and lack of outcome indicators. In addition, no relevant studies that met the inclusion criteria were found in the references of the included literature, and although the literature that met the criteria was found in the guidelines, this part of the literature has been detected in the early stage of systematic retrieval, and no new inclusion literature supplement has been formed. Finally, a total of 24 randomized controlled trials (RCTs) were included for analysis.

### Characteristics of included literature

The characteristics of the 24 studies included in the final analysis are shown in **Supplementary Table 1**. A total of 2,352 patients with DFU were included in the literature, with 15 categories of interventions containing single non-pharmacological interventions and multi-measure combinations, with publication dates ranging from 2000 to 2024. 23 studies published in English (15, 17, 19–22, 25, 26, 28, 30–36, 38–44), 1 published in Chinese (45). There are 5 studies conducted transnationally (20, 21, 25, 26, 28), 9 in the United States (15, 19, 22, 30, 31, 34, 38, 42, 44), 1 in Israel (17), 4 in India (32, 33, 36, 39), 1 in Mexico (35 ), 2 in China (43, 45), 1 in Egypt (41), 1 in Turkey (40). Study Design No study was triple-blind, 7 double-blind (17, 20–22, 25, 26, 31), 7 single blind (15, 28, 30, 32, 34, 35, 38). The rest did not involve a blind method. There was only one three-arm randomized controlled trial (3-armed RCT) (43), and the rest were two-armed trials. The sample size of the single study was the least (15/15) (41), and the most (172/164) (20). The intervention time was 2, 4, 8, and 12 weeks. There were 22 articles reporting 12-week healing rate (15, 17, 19–22, 25, 26, 28, 30–34, 36, 38–40, 42–45), and 8 articles reporting healing time (26, 30, 33–35, 39, 41, 43).

Baseline characteristics of the included population: A total of 23 studies that explicitly reported gender data were included. The intervention group (IG) and the control group (CG) included a total of 2352 people, of which 70.76% (1679/2352) were males and 29.24% (694/2352) were females. Another study of gender data was not reported (38), not included in the above statistics. The age range of the included population was 19–89 years old (only 1 reported the age range (26), and the remaining studies reported the average age). Among the 23 studies, the average age of the intervention group and the control group was between 48 and 67 years old, and the overall average age was about 58.7 years old. The DFU grades included in the study were predominantly mild to moderate ulcers (Wagner Grades 1-2, University of Texas Grades 1A/1C) (60% of the population, 14/23), and moderately severe ulcers (Wagner Grades 2-3/2-4, University of Texas Grades 2-3) in 30.4% of the population (7/23); One of the non-standardized grading studies was conducted (19), However, considering the similarity of their baseline characteristics with other studies and the clear reference to the ulcer site and good circulatory status, which excludes the possibility of Wagner Grade 0 or 5, it was finally agreed, after a two-person discussion, that they could be included in the review. The 12-week healing rate of the intervention group was up to 93.2% (45), and the lowest was 20% (40). The 12-week healing rate of the control group was up to 53.8% (32), and the lowest was 3% (40). The shortest healing time of the intervention group was 14.82 ± 7.30 days (33), and the longest was 78.19 ± 19.11 days (39). The shortest healing time of the control group was 44.57 ± 9.29 days (33), and the longest was 101.5 ± 62.3 days (35).

### Quality evaluation

**Figure 2:**
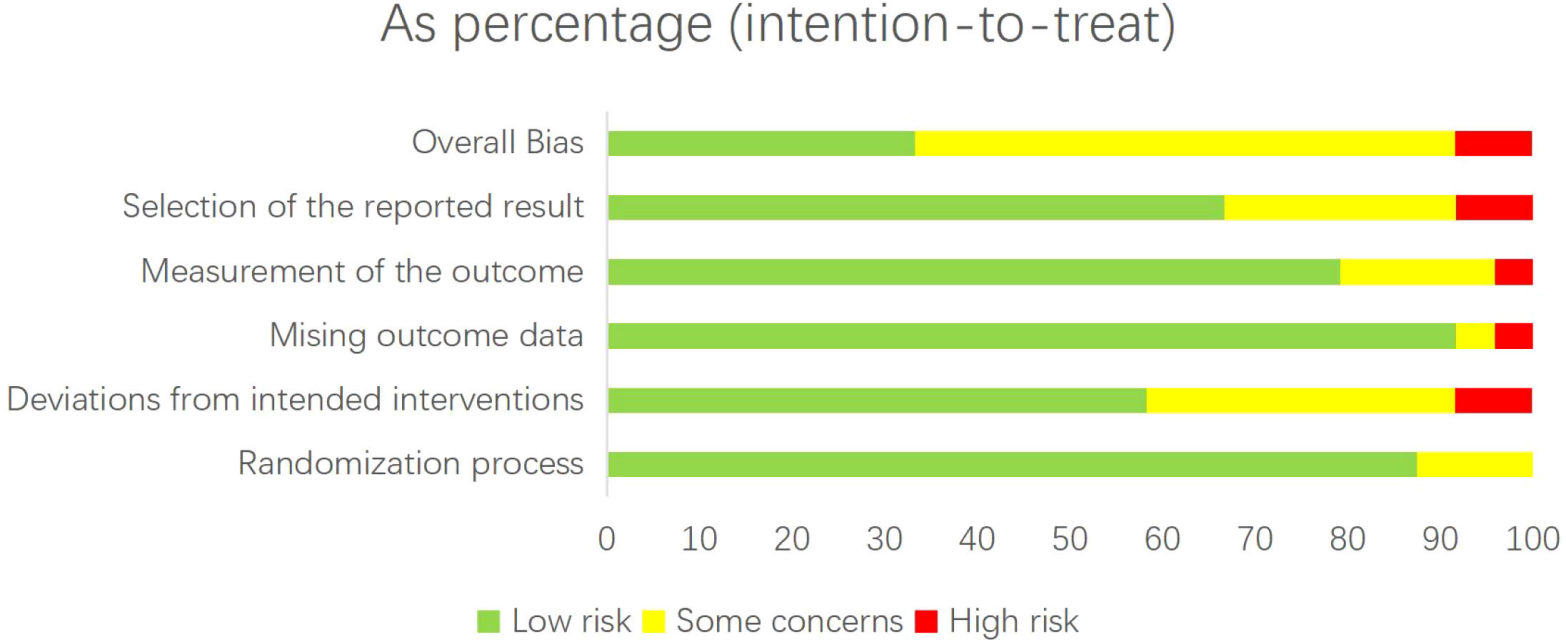
Risk of bias of the selected literature assessed by RoB (version 2).

After quality assessment of the 24 studies included in the NMA, it was found that 8 studies were low risk (15, 20–22, 25, 33, 38),2 were high risk (17, 40), and 14 had certain concerns (19, 28, 30–32, 34–36, 39, 41–45). During the randomization process, three of them were rated as having certain concerns, because they did not describe specific allocation concealment methods (39, 40, 45). In terms of deviation from the established intervention measures, 8 were rated as having some concerns (19, 28, 31, 32, 34–36, 42), because some people were aware of the grouping. 2 of them were rated as high risk (30, 40), because there were more patients lost to follow-up and no blind method was implemented. In terms of missing outcome data, one was rated as having some concerns (42), because the missing data processing method was not described. One was rated as high risk (17), because of the high rate of data loss leading to inter-group imbalance. In the measurement of outcome indicators, 4 were rated as having some concerns (35, 41, 42, 44), because the outcome evaluator knows the grouping, but the outcome is an objective indicator. One item was rated as high risk (40), because blinding was not used and there were multiple measurement time points. In terms of analysis scheme and result selection, 6 items were rated as having certain concerns (35, 36, 41, 43–45), because it was not explicitly mentioned whether there was a preset analysis scheme, but no selective report was found. There are two high risks (17, 40) because they are clearly not analyzed according to the preset scheme. The risk bias visualization of the included literature is presented in **Figure 2**.

### Traditional meta-analysis

**Figure 3:**
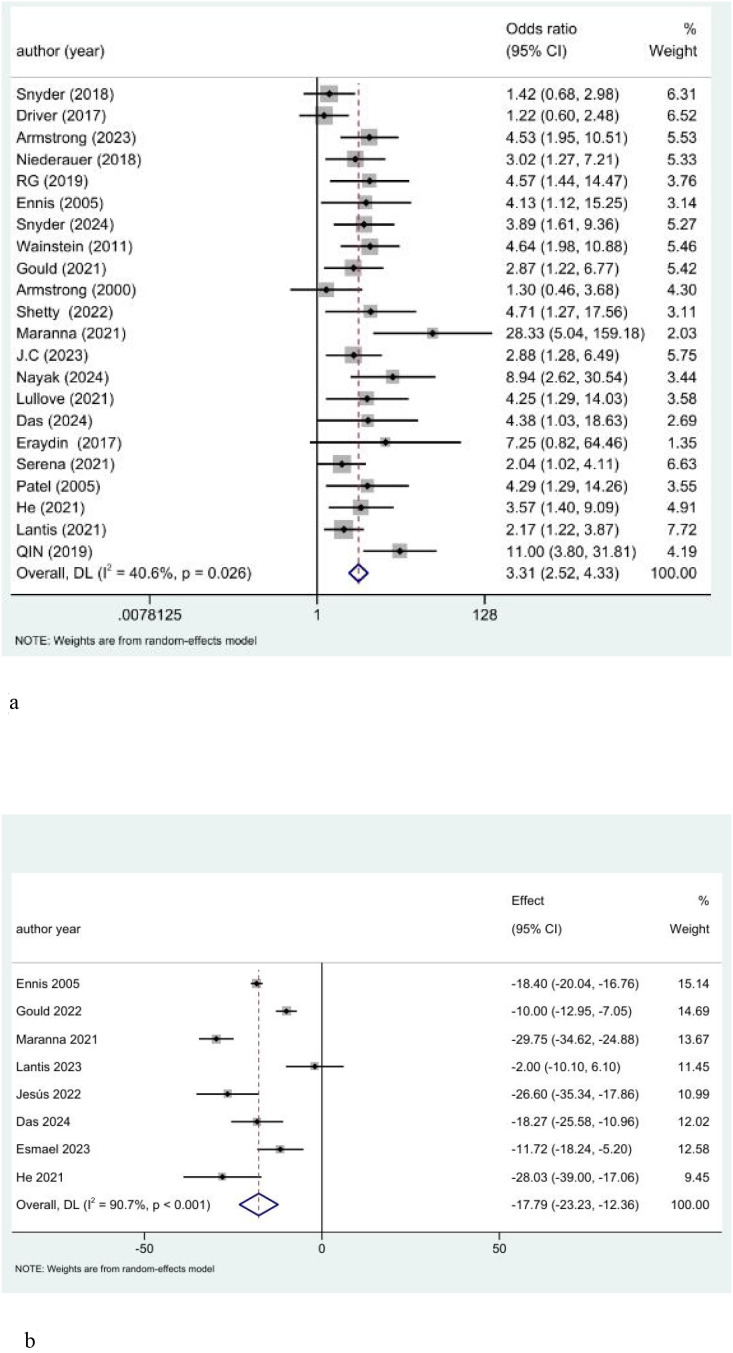
Findings of the traditional meta-analysis. **(a)** Forest plot of the 12-week healing rate. **(b)** Forest plot of the ulcer healing time.

In the study of the 12-week healing rate, by observing the *I (*2*)* statistic (*I ^2^* = 40.6%, *P* = 0.026 < 0.05), it was found that there was heterogeneity but within the acceptable range. The detailed results are shown in **Figure 3**. In order to make the results more conservative, we still use the random effect model to merge. The results showed that compared with the control measures, non-drug intervention significantly increased the 12-week healing rate of DFU (OR = 3.31, 95% CI: 2.52 to 4.33).

In the study of healing time, significant heterogeneity was observed through the *I ^2^* statistic (*I ^2^* = 90.7%, *P* < 0.001), and the detailed results are shown in **Figure 3**. Therefore, we use the random effect model to merge. The results showed that compared with the control measures, non-drug intervention significantly reduced the ulcer healing time (MD = -17.79, 95% CI: -23.23 to -12.36). We accept the heterogeneity between different studies and classify direct pairwise comparisons between different types of interventions.

After the classification, we will re-*I ^2^* test for studies greater than or equal to 2, which can be seen in **Supplementary Figure 1**. In the study of the 12-week healing rate, there were 5 items of GT + SC *vs* SC, and a small heterogeneity was observed through *I ^2^* statistics (*I ^2^* = 46.4%, *P* = 0.114 > 0.05), within an acceptable range. There were two items of allograft skin graft (AS) + SC *vs* SC, and no significant heterogeneity was observed (*I ^2^* = 0%, *P* = 0.960 > 0.05). There were two items in AS + SC *vs* DT + SC (*I ^2^* = 63.1%, *P* = 0.100 > 0.05), and there was heterogeneity in the same direction, that is, similar intervention measures, which were significantly different from the control group, but the difference in effect size between the two groups was significant. Through discussion, we found that one of the studies (You 2014) (18), has a high-risk quality bias in blinding (evaluators do not blind) and analysis plans (unregistered analysis programs), which may be exaggerating the actual effect size and ultimately considering not to include this study. There were 2 items of Xenogeneic Skin Grafts (XSG) + SC *vs* DT + SC (*I ^2^* = 0%, *P* = 0.598 > 0.05), and there was no heterogeneity. There were 2 items of ET + SC *vs* SC (*I ^2^* = 0%, *P* = 0.869 > 0.1), and there was no heterogeneity. There are 2 items of Autologous Blood-Derived Products (ABDP) + SC *vs* SC (*I ^2^* = 0%, *P* = 0.891 > 0.1), and there is no heterogeneity.

In the analysis of healing time, the overall combination showed extremely high heterogeneity (*I ^2^* = 90.7%). After verification, each of the eight studies assessed eight completely different interventions in comparison (i.e., eight different direct comparisons). However, substantial heterogeneity remained for healing time (I²>80%). Exploratory subgroup analyses based on ulcer type, treatment duration, RoB 2 overall risk of bias, country, blinding, control type and publication year did not materially reduce heterogeneity (all subgroup I² values remained >80%; data not shown). Since each comparison group contained only one study, the heterogeneity within the group could not be evaluated. Therefore, we directly reported the combined results of the random effects model and were cautious about the interpretation of the results in the discussion.

### Results of network meta-analysis

**Figure 4:**
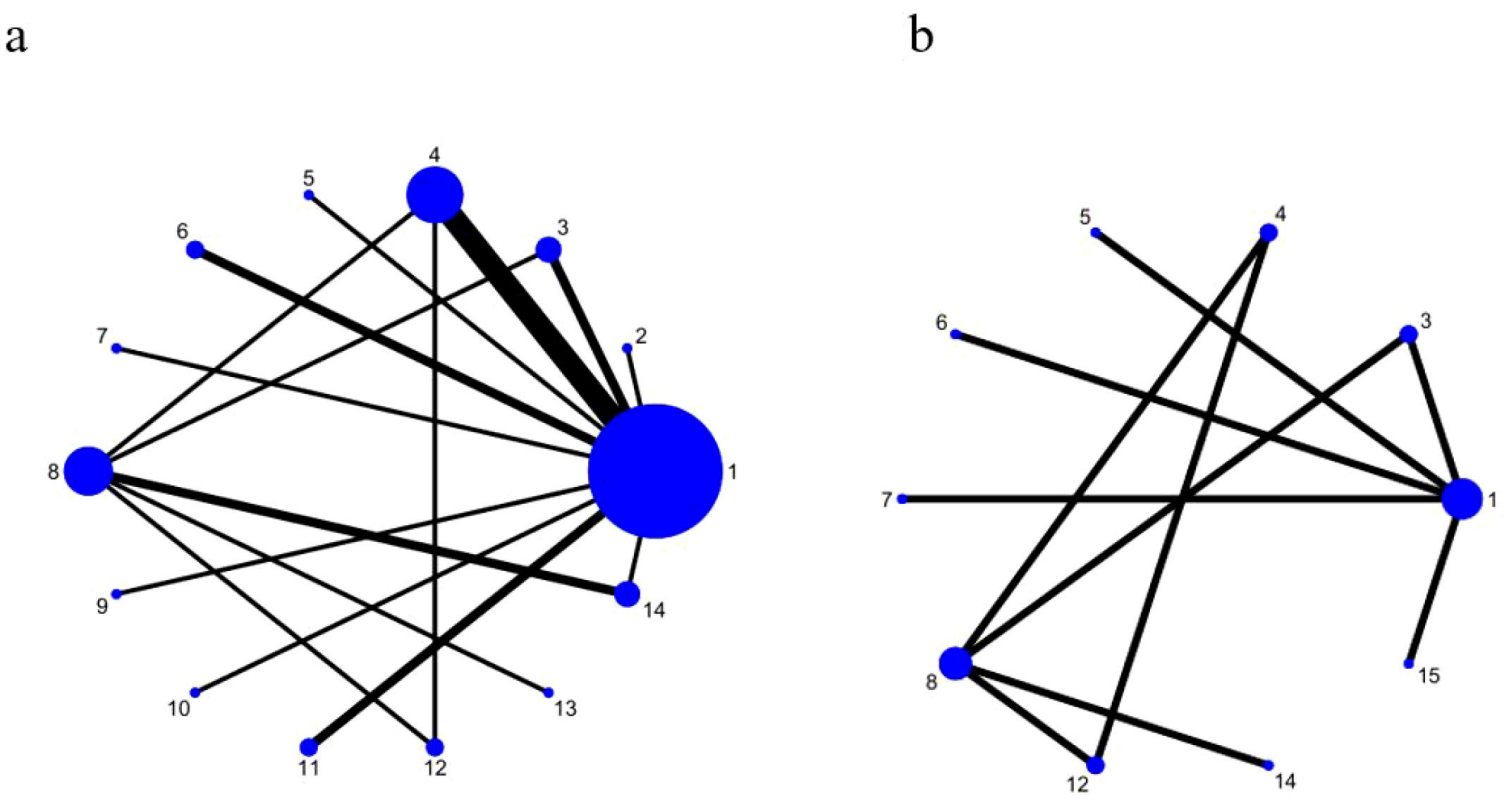
Network plot. **(a)** the 12-week healing rate. **(b)** the healing time. 1 = Standard Care or Standard Care + Placebo; 2 = Standard Care + Focused Extracorporeal Shock Wave Therapy; 3 = Standard Care + Allograft Skin; 4 = Standard Care + Gas Therapy; 5 = Standard Care + Ultrasound Therapy; 6 = Standard Care + Autologous Blood-Derived Products; 7 = Standard Care + Negative Pressure Wound Therapy; 8 = Standard Care + Dressing Therapy; 9 = Standard Care + Pneumatic Therapy; 10 = Standard Care + Non-contact Normal-temperature Wound Therapy; 11 = Standard Care + Exercise Therapy; 12 = Standard Care + Gas Therapy + Dressing Therapy; 13 = Standard Care + Autologous Blood-Derived Products + Dressing Therapy; 14 = Standard Care + Xenogeneic Skin Grafts; 15 = Standard Care + Phototherapy.

The NMA included 15 interventions involving a total of 2352 DFU patients. For the meaning of each intervention, see **Supplementary Table 2**. **Figure 4** shows the network weights for the comparison of eligible treatment effects. The results of NMA are shown in **Supplementary Table 3**.

### Network evidence diagram

The network diagram can visualize various intervention measures. In terms of the 12-week healing rate, all included studies had a total of 15 interventions, forming multiple closed loops. In the network graph, the larger nodes indicate that there are more sample sizes. The network graph shows that the nodes of SC are the largest, indicating that the sample size is the largest. The thicker lines between nodes represent more comparisons between interventions. The line of comparison between SC and SC + GT was the thickest, indicating that the number of studies comparing these two interventions was the largest. Specifically, in 22 studies, there were 17 items with SC as the control group (15, 17, 19–22, 25, 26, 28, 31–33, 36, 39, 40, 42, 44), and 5 items using SC + DT as the control group (30, 34, 38, 43, 45). In the intervention group, 5 were treated with SC + GT (17, 21, 22, 25, 42), 3 with SC + AS (15, 30, 32), 3 with SC + ABDP (28, 39,43 ), 2 with SC + Exercise Therapy (ET) (36, 40), and 3 with SC + XSG (34, 38, 44). Each one was treated with SC + Focused Extracorporeal Shock Wave Therapy (ESWT) (20), SC + Ultrasound Therapy (UT) (26), SC + NPWT (33), SC + Pneumatic Therapy (PT) (31), SC + Non-contact Normal-temperature Wound Therapy (NNWT) (19), SC + GT + DT (43), SC + ABDP + DT (45).

In terms of healing time, all included studies had a total of 10 interventions, and only one closed loop was formed by the three-arm experiment (43). The sample size of SC is the largest. The number of studies comparing each of the two interventions is 1. Among them, there were 6 items with SC as the control group (26, 33–35, 39, 41), and 2 items with DT + SC as the control group (30, 43). In the intervention group, there were two items using SC + AS (30, 35), each item was used separately SC + UT (26), SC + NPWT (33), SC + XSG (34), SC + ABDP (39), SC + P (41), SC + GT + DT (43).

### Evaluate statistical consistency

In the 12-week healing rate, the global consistency of each type of intervention was consistent with the consistency hypothesis (Chi square = 1.72, P = 0.4226 > 0.05). The healing time was also (Chi square = 0.23, P = 0.6283 > 0.05). It shows that there is no significant global inconsistency between direct estimation and indirect estimation, that is, indirect estimation is comparable to direct evidence (**Supplementary Figure 2**). The inconsistency of each node is analyzed by the node splitting method. The results showed that the P value > 0.05, there is no inconsistency, and the results of direct comparison and indirect comparison could be combined. In the 12-week healing rate, three closed loops were found, and the consistency of the loops was consistent with the consistency hypothesis (P > 0.05). In particular, in the healing time intervention, there is only one closed loop, and it consists of three-arm experiments, so there is no inconsistency in the loop.

### Ranking of interventions

**Figure 5:**
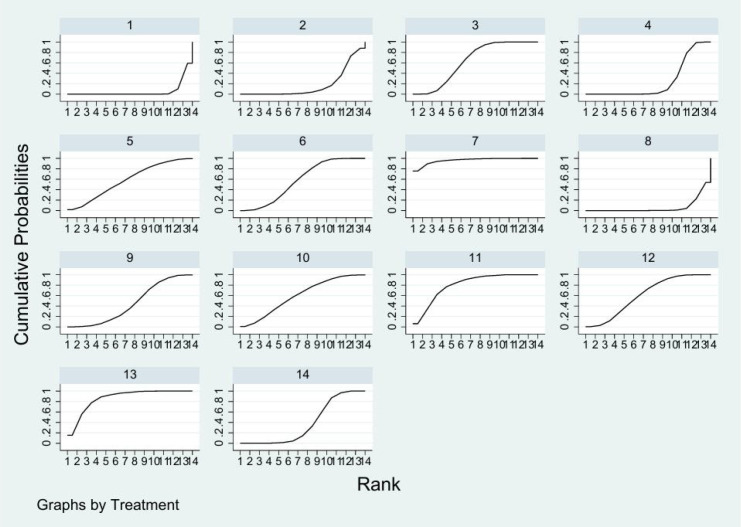
Cumulative probability plots. 1 = Standard Care or Standard Care + Placebo; 2 = Standard Care + Focused Extracorporeal Shock Wave Therapy; 3 = Standard Care + Allograft Skin; 4 = Standard Care + Gas Therapy; 5 = Standard Care + Ultrasound Therapy; 6 = Standard Care + Autologous Blood-Derived Products; 7 = Standard Care + Negative Pressure Wound Therapy; 8 = Standard Care + Dressing Therapy; 9 = Standard Care + Pneumatic Therapy; 10 = Standard Care + Non-contact Normal-temperature Wound Therapy; 11 = Standard Care + Exercise Therapy; 12 = Standard Care + Gas Therapy + Dressing Therapy; 13 = Standard Care + Autologous Blood-Derived Products + Dressing Therapy; 14 = Standard Care + Xenogeneic Skin Grafts.

The interventions are listed according to their effectiveness ranking in the cumulative probability map (**Figure 5**) and the SUCRA value. According to the results of SUCRA, SC + NPWT was rated as the highest-ranked intervention (96.1%) in terms of 12-week healing rate. Followed by SC + ABDP + DT (86.5%), SC + ET (80.4%), SC + AS (63.3%), SC + GT + DT (61%), SC + NNWT (60%), SC + UT (58.1%), SC + ABDP (57.7%), SC + PT (44.9%), SC + XSG (38.3%), SC + GT (24.7%), SC + ESWT (17.6%), SC + DT (6.3%) and SC (5.4%). In terms of healing time, SC + GT + DT (100%) was the highest ranking intervention, followed by SC + XSG (79%), SC + GT (75.5%), SC + DT (74.1%), SC + NPWT (56%), SC + AS (46.7%), SC + UT (28.2%), SC + ABDP (28%), SC + P (12.4%), SC (0%).

### Publication bias and sensitivity analysis

**Figure 6:**
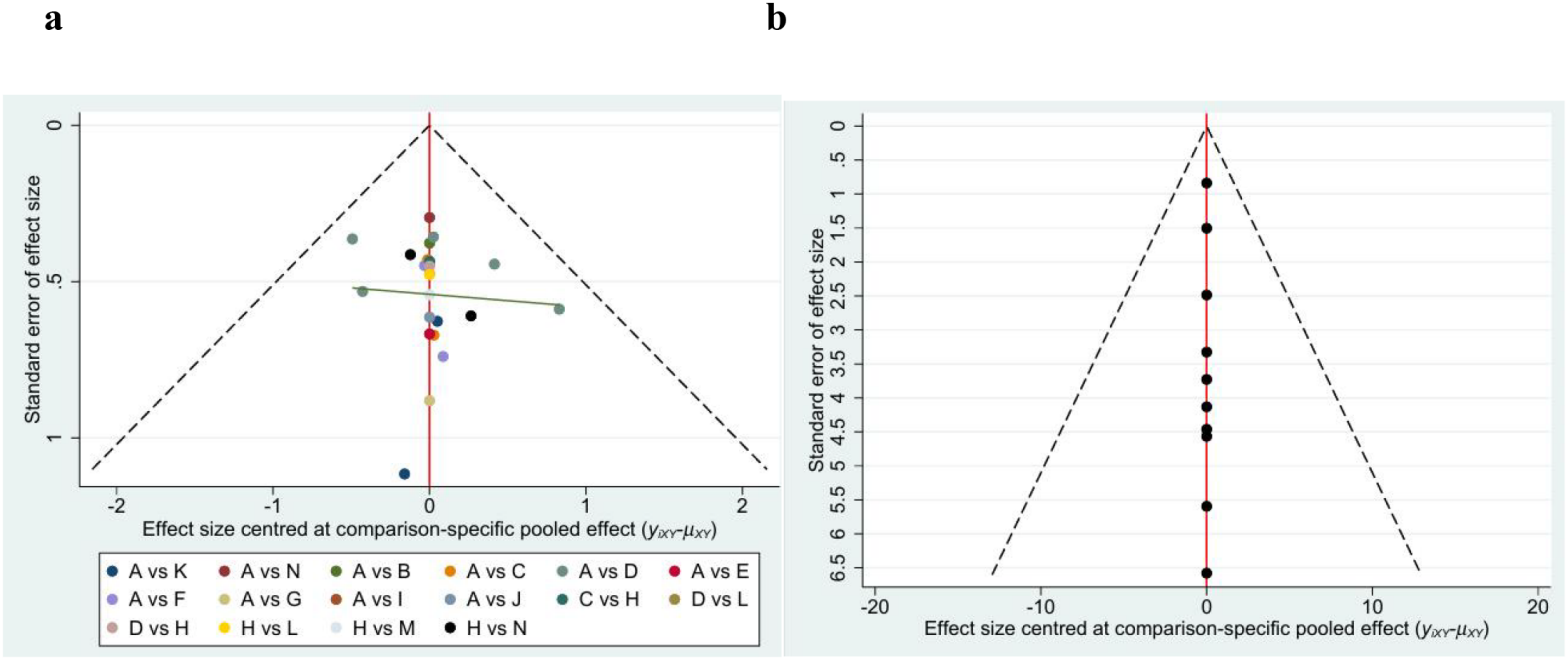
The funnel plot. A=Standard care or Standard Care + Placebo; B=Standard Care + Focused Extracorporeal Shock Wave Therapy; C= Standard Care +Allogeneic Skin Grafts; D= Standard Care + Gas Therapy; E= Standard Care + Ultrasound Therapy; F= Standard Care + Autologous Blood-Derived Products; G= Standard Care + Negative Pressure Therapy; H= Standard Care + Dressing Therapy; I= Standard Care + Pneumatic Therapy; J=Standard Care + Non-contact Normal Temperature Wound Therapy; K= Standard Care + Exercise Therapy; L= Standard Care + Gas Therapy + Dressing Therapy; M= Standard Care + Autologous Blood-Derived Products + Dressing Therapy; N=Standard Care + Xenogeneic Skin Grafts; O= Standard Care + Phototherapy.

The corrected comparison funnel plot shown in **Figure 6** shows a nearly symmetrical scatter distribution, which visually suggests a low risk of publication bias. However, it should be noted that the visual judgment of the funnel plot is subjective, and it may not be able to accurately identify the subtle asymmetry of the effect size distribution of small sample studies. For further verification, we performed the Egger test and Begg test on 22 studies related to the 12-week healing rate and found significant publication bias (*P* < 0.05), indicating that potential publication bias may significantly affect the interpretation of the results. The Egger test and Begg test showed that there was no significant publication bias (*P* > 0.05) in 8 studies related to healing time, suggesting that there may be no publication bias. All pairwise comparison interventions for the two outcomes were analyzed for sensitivity by excluding each study in turn, and the results were consistent with the results obtained before exclusion (**Supplementary Figure 3**).

## Discussion

### Network meta-analysis

The purpose of this study was to evaluate the efficacy of different non-drug interventions in DFU healing. To this end, we used NMA to quantify the efficacy of 15 types of non-drug interventions in 24 RCTs (2352 patients) included. Detailed definitions and core mechanisms of these interventions are provided in **Supplementary Table 2**. In terms of 12-week healing rate, NPWT + SC had the highest efficacy point estimation and optimal ranking (OR = 28.33, 95% CI: 5.04 to 159.18, SUCRA = 96.1%). However, its 95% confidence interval range is extremely wide (5.04-159.18), indicating that there is a high degree of uncertainty in the true effect size, which may range from a clinically significant but non-extreme advantage to a very large advantage. Therefore, although SUCRA ranked NPWT + SC as the top intervention for the 12-week healing rate, this ranking is driven by a single small trial with a very wide confidence interval and should be interpreted with great caution. The SUCRA values in this study should be considered exploratory indicators of relative efficacy rather than definitive evidence of superiority. Consequently, a large sample of RCTs is needed in the future, especially for direct comparison with other highly effective interventions (such as ABDP combined with DT + SC) to verify the relative efficacy and accurate estimation of the effect size. ABDP + DT + SC was the second (OR = 10.93,95% CI: 3.29,36.32, SUCRA = 86.5%). There were some concerns about the quality of the study. Without a specific description of allocation concealment and blinding, the efficacy may be overestimated. In terms of healing time, the GT + DT + SC combined intervention had the most significant effect (MD = -64.60, 95% CI: -78.92, -50.27, SUCRA = 100%). However, this conclusion was only based on one study, and there were some concerns about the quality of the study. The outcome measurer did not implement blinding, which may overestimate the efficacy. Given that each comparison for healing time was informed by only one study, these probability rankings cannot be used alone to guide clinical decision-making.

### Traditional meta-analysis

Overall, non-pharmacological interventions significantly improved the 12-week ulcer healing rate compared to control measures (OR = 3.31, 95% CI: 2.52 to 4.33). In the GT + SC *vs* SC study, there was a small heterogeneity (*I^2^* = 46.4%). This is consistent with the existing research (49), except for the significant difference between hyperbaric oxygen and some therapies at 12 weeks, there was no significant difference in the efficacy of other GT, and the effect direction was consistent, thus reducing the heterogeneity. According to the heterogeneity within the acceptable range and the conclusion of this study, we do not consider subgroup analysis.

In terms of healing time, although the combined results showed an average shortening of about 17.79 days (MD = -17.79, 95% CI: -23.23 to -12.36), the heterogeneity was extremely high (*I^2^* = 90.7%). To explore this,exploratory subgroup analyses based on ulcer type, treatment duration, RoB 2 overall risk of bias, country, blinding, control type, and publication year were conducted; however, these did not materially reduce the heterogeneity (all subgroup *I* ² values remained > 80%). This sustained high heterogeneity likely stems from the huge intrinsic differences in the efficacy of various non-pharmacological interventions themselves. Furthermore, because healing time is often evaluated as a secondary outcome, the measurement methods and follow-up endpoints were not entirely consistent across trials. It is also crucial to acknowledge the fragile evidence structure for this outcome: the included studies evaluated 7 different interventions across 8 RCTs, where only one intervention was supported by 2 RCTs, and the remaining 6 interventions were each supported by only a single small-sample RCT.This sparse network makes it impossible to effectively evaluate the source of heterogeneity within the group. The extremely high *I* ² value suggests that there are huge differences in the effect of different non-drug interventions on shortening the healing time (69). Because of this, it also supports NMA for these 8 studies,nevertheless, given the weak evidence base, the NMA results for healing time must be interpreted strictly as exploratory. They serve primarily to propose potentially promising intervention directions for future research, rather than definitive clinical recommendations.

### Evidentiary value

Clinical medical staff can choose the treatment plan according to the treatment goal. When the treatment goal is to improve the 12-week healing rate, NPWT + SC can be given priority. GT + DT + SC hints at potential advantages when the goal is to quickly shorten the healing time. The above selection should be combined with ulcer severity, ischemia/infection status, and accessibility. It should be considered that the treatment costs of NPWT and ABDP + DT are high, and patients with limited economic affordability may not accept these two interventions. However, one study shows that although the initial cost of NPWT therapy is higher than that of traditional dressing change, it can improve the healing rate and ultimately reduce the economic burden as a whole by accelerating healing and reducing long-term benefits such as amputation (70). In view of this, if the wound is refractory, the area is large, and the Wagner Grade is 3-4, patients with DFU who may need long-term treatment may choose NPWT. If the wound is primary, the area is small, or the Wagner Grade is 1-2, and the patient’s economic conditions are poor, the SC + ET intervention model can also be used. It needs to be stated that the ranking results of this study cannot be equated with the absolute difference in the efficacy of interventions. The lower-ranked interventions are not ineffective, and the potential of their combined application needs to be confirmed by more high-quality RCTs. In clinical practice, it can be used as an auxiliary method combined with other interventions to provide patients with more flexible treatment options. Finally, regardless of the intervention mode selected by the patients, the medical staff needs to inform the estimated efficacy and overall cost of each type of intervention in advance.

### Similarities and differences with previous network meta-analysis

#### Research design

PRISMA ‘s expanded statement points out the limitations of review studies that generally have heterogeneity in the definition of patient population, treatment plan, and outcome (59). But this NMA is more stringent in the definition of the population, and it is clearly required that the inclusion study must report and limit the DFU to Wagner Grades 1–4 or Texas Grades 1-3 (excluding Wagner Grade 0,5 or the same type of classification), in order to minimize the clinical heterogeneity introduced by the inclusion of high-risk feet without wound intervention or gangrenous feet requiring emergency surgery. At the end of this study, a non-graded DFU study was included (19). Considering that the study fully illustrated the plantar ulcer with good blood circulation, similar to Wagner Grades 1-2, and the lack of such measures, it was included after discussion by two people. This point can be referred to in future studies, that is, if there is a lack of research samples, the inclusion criteria can be considered to be relaxed.

#### Research results

In this study, NPWT + SC was the most effective non-drug intervention in the 12-week healing rate of DFU, followed by SC + ABDP + DT. It is noteworthy that this conclusion is consistent with the trend of previous NMA results (50). Previous research results show that NPWT combined with platelet-rich plasma (PRP) ranked first in the complete healing rate of DFU (OR = 22.0, 95% CI: 2.5 to 23.0), and compared with NPWT alone, the combined regimen had a significant advantage in healing (OR = 3.5, 95% CI: 1.20 to 10.0). However, it should be noted that the time nodes of the five original studies included in the NMA are not uniform (2–6 weeks), and the lack of a standardized time reference may have an impact on the comparability of the results. Another NMA showed that stem cell therapy had the best healing effect (OR = 5.71, 95% CI: 2.64 to 12.34), while NPWT alone only ranked sixth (50). The core reason for this difference may be that the study does not limit the type of intervention and includes drug measures such as growth factors; its top stem cell therapy, amniotic membrane therapy, and other interventions were not included in the comparative category of non-drug intervention in this study. Nevertheless, the efficacy of non-drug measures such as oxygen therapy and low-frequency ultrasound in that study was second to that of NPWT, which was highly consistent with the results of this study.

In addition, an NMA pointed out that hyperbaric oxygen therapy (HBOT) and ESWT need to be continuously treated for more than 12 weeks to show a clear effect, which further confirms the scientificity and rationality of 12 weeks as the evaluation node of healing rate in this study (51).

In terms of shortening the healing time of DFU, the results of this study showed that the combined non-drug intervention of GT + DT + SC had the best effect in the included comparison regimen (MD = -64.60, 95% CI: -78.92 to -50.27, SUCRA = 100%). However, it should be pointed out objectively that there is no research consistent with this result. An NMA on the efficacy of different gases on DFU pointed out that there is insufficient evidence to confirm that a certain GT has a clear effect on shortening the healing time of DFU and is superior to other therapies (49).

### Limitations

#### Research level: risk of bias and insufficient population representativeness

Of the 24 RCTs included in this study, only 33.3% (8/24) achieved a low risk of bias, and 58.3% (14/24) had certain concerns: Non-standard randomization process (no description of allocation concealment) and insufficient implementation of blinding (although individual interventions are too different to blind between implementers and patients, the most important evaluators do not use blinding) may lead to overestimation of the primary outcome (100% epithelization). The missing rate of outcome data in 2 high-risk studies was more than 20% and the treatment method was not explained, which further weakened the reliability of the results. There was a significant imbalance in group characteristics: males accounted for 70.76% (1679/2352), and female data were scarce. Clinical studies suggested that the degree of neuropathy and wound healing rate of female DFU patients may be different from those of males, resulting in limited extrapolation of results to female groups. It is recommended that more female samples should be included in future studies.

#### Evidence network level: the scarcity and transitivity challenges of direct comparison

In the study of 12-week healing rate, after classifying the studies according to the comparison between interventions, it was found that there was a small heterogeneity within GT + SC *vs* SC (*I^2^* = 46.4%), and no subgroup analysis was performed, which may have a potential impact on the transitivity of indirect evidence. In addition, although the global consistency test showed that there was no contradiction between direct and indirect evidence (*P* > 0.05), multiple interventions such as ESWT + SC and NNWT + SC were only supported by one study, and their efficacy ranking was completely dependent on indirect evidence, and the accuracy of the effect size was insufficient. In addition, due to the small sample size of NPWT + SC core intervention, the uncertainty of the effect size of healing rate (OR = 28.33, 95% CI: 5.04 to 159.18) is extremely high, and the true clinical benefit range cannot be accurately judged. There were only 8 included studies related to healing time, and the scope of intervention measures involved was narrow. Many potential non-drug interventions (such as ESWT, ABDP + DT, etc.) were not included in the direct or indirect comparison of the outcome. This situation is mainly due to the fact that this study has set strict standards, such as standardized DFU staging and a clear definition of outcomes for the included literature, resulting in an insufficient number of documents that meet the requirements, which in turn makes the evidence network imperfect and needs to avoid excessive promotion of this conclusion.Overall, the sparse network structure, the reliance on indirect evidence for several highly ranked interventions, and the detected publication bias mean that the SUCRA rankings should be interpreted as hypothesis-generating and should not be over-interpreted as definitive treatment recommendations.

#### Overview level: publication bias and limitations of outcome indicators

For the 22 studies of 12-week healing rate, although the corrected comparative funnel plot showed an approximately symmetrical scatter distribution visually, which seemed to suggest a lower risk of publication bias, both the Egger test (*P* < 0.05) and the Begg test (*P* < 0.05) showed significant publication bias. This contradiction stems from the inherent difference between the two evaluation methods: the visual judgment of the funnel plot is subjective, and it is difficult to identify the subtle asymmetry of the effect size distribution in small sample studies, which may overestimate the actual efficacy of efficient interventions. In terms of outcome indicators, DFU has high recurrence characteristics, but studies that meet the inclusion criteria rarely mention the recurrence rate, so there is a lack of comparative evaluation of the recurrence rate. Furthermore, according to the GRADE assessment, the certainty of evidence for most key comparisons was rated as low to very low, mainly driven by risk of bias, inconsistency, imprecision, and publication bias (see **Supplementary Table 4**). Therefore, the results of this study should be interpreted with caution.

## Conclusion

In terms of 12-week healing rate, NPWT + SC may be the best, followed by ABDP + DT + SC, and ET combined with SC also showed good results. Clinical medical staff can choose appropriate intervention measures according to individual conditions, such as ulcer grade and the economic status of patients. In terms of shortening ulcer healing time, although the combined intervention of GT + DT + SC showed the most significant effect, given that this conclusion is supported by only individual studies and there is potential bias in the outcome evaluation method, the results of this study should be viewed as a preliminary ranking of promising non-pharmacological interventions, rather than a definitive clinical reference for the “best treatment regimen”. Therefore, caution is needed in clinical application, and further verification by more high-quality studies is required.

## Data Availability

The original contributions presented in the study are included in the article/**Supplementary Material**. Further inquiries can be directed to the corresponding authors.
