# Supplementary material for: Effects of non-pharmacological interventions on ulcer healing in patients with diabetic foot: a network meta-analysis of randomized controlled trials
Source: Front Endocrinol (Lausanne). 2026 Mar 26;17:1811595. doi: 10.3389/fendo.2026.1811595 (PMC13061723; doi:10.3389/fendo.2026.1811595)

**Supplementary Fig. 1. Heterogeneity test after classification**

**12-week healing rate**

**1.** **Gas Therapy + Standard Care vs Standard Care**


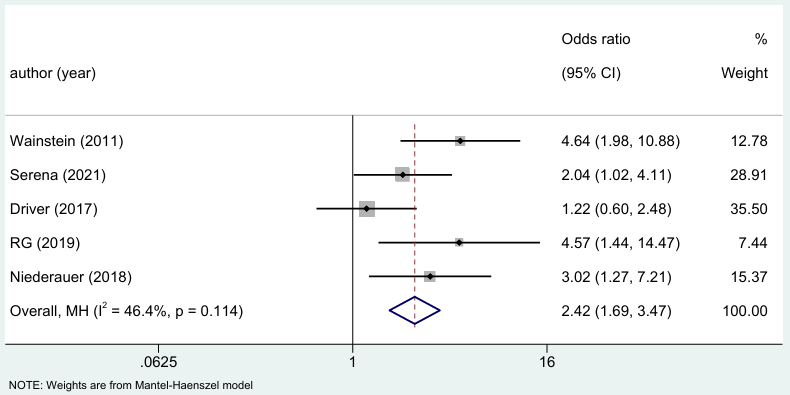


**2.** **Allograft Skin + Standard Care vs Dressing Therapy+ Standard Care**


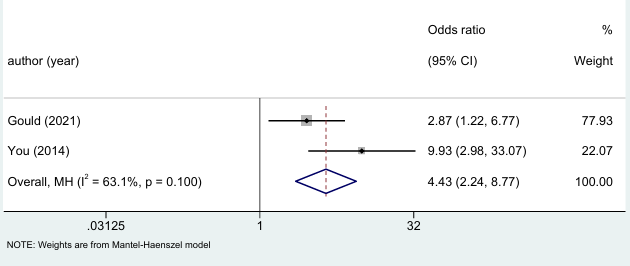


**3. Allograft Skin + Standard Care vs Standard Care**


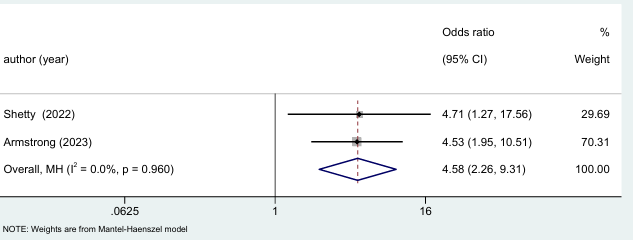


**4. Xenogeneic Skin Grafts + Standard Care vs Dressing Therapy+ Standard Care**


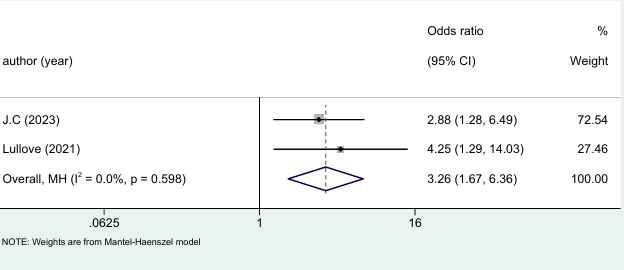


**5. Exercise Therapy + Standard Care vs Standard Care**


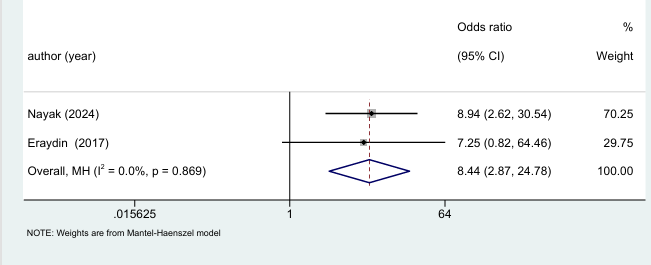


**6. Autologous Blood-Derived Products + Standard Care vs Standard Care**


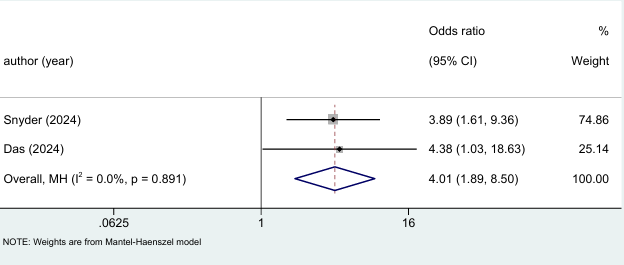

Supplement: Supplementary file 1 [file DataSheet1.docx]
