# Supplementary figures and images for: Effects of non-pharmacological interventions on ulcer healing in patients with diabetic foot: a network meta-analysis of randomized controlled trials
Source: Front Endocrinol (Lausanne). 2026 Mar 26;17:1811595. doi: 10.3389/fendo.2026.1811595 (PMC13061723; doi:10.3389/fendo.2026.1811595)

**Supplementary Fig 2. Global inconsistencies**

**(a)** **12-week healing rate**


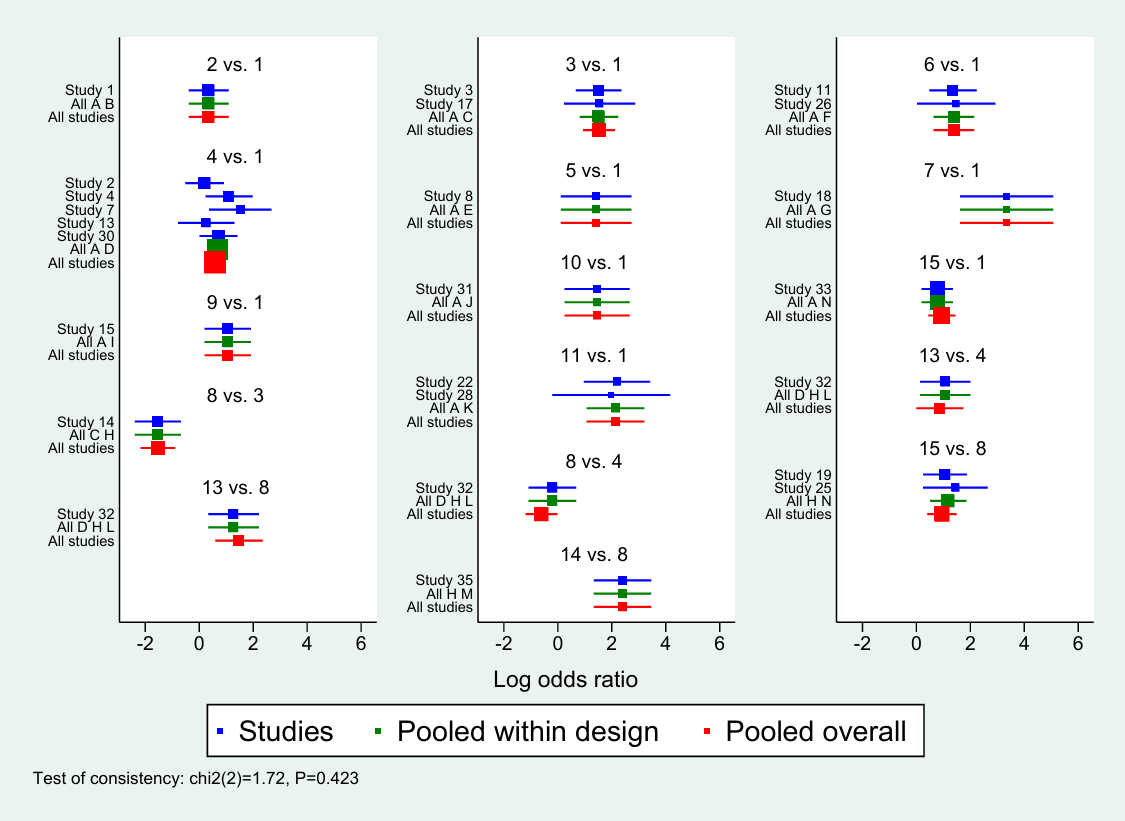


**(b) healing time**


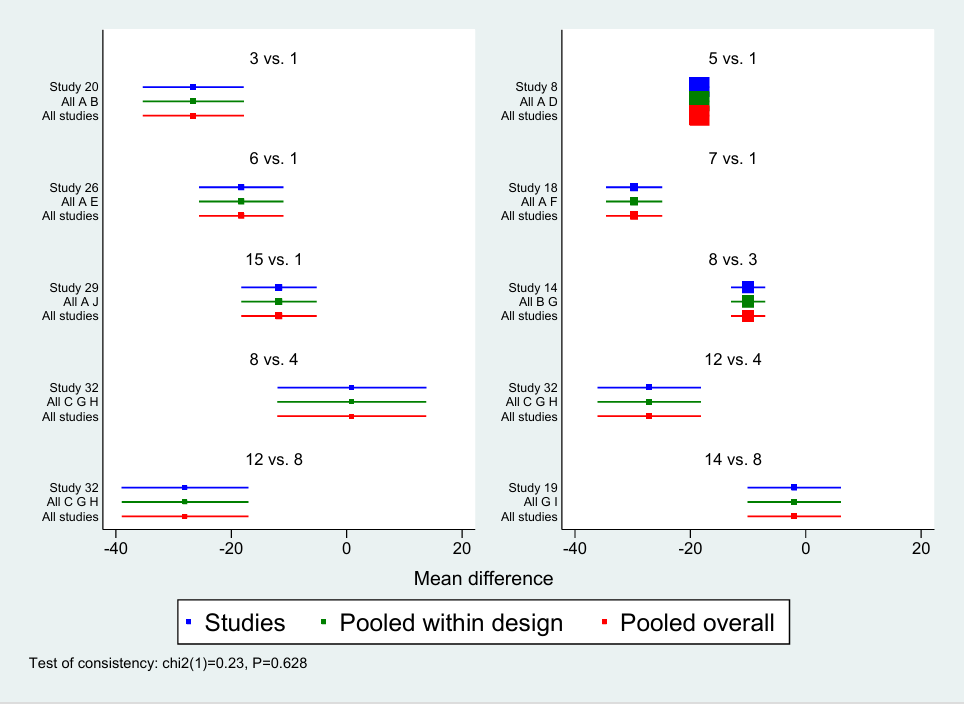

Supplement: Supplementary file 2 [file DataSheet2.docx]

**Supplementary Fig. 3** sensitivity analysis

(a) 12-week healing rate


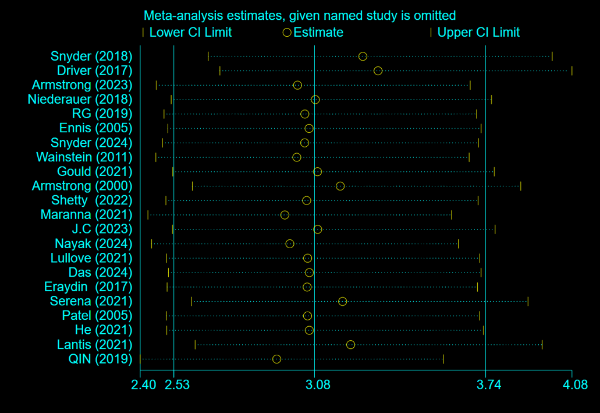


(b) healing time
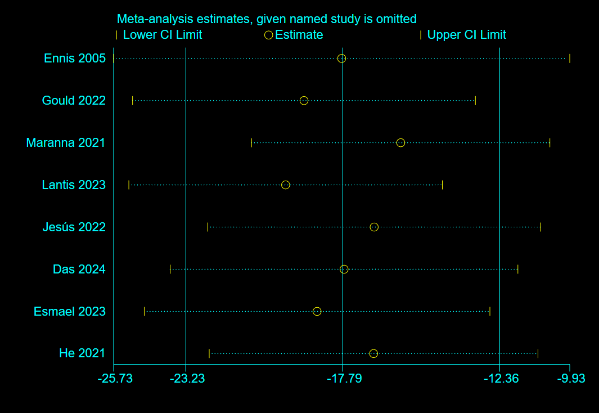

Supplement: Supplementary file 3 [file DataSheet3.docx]
