# Supplementary material for: Effects of non-pharmacological interventions on ulcer healing in patients with diabetic foot: a network meta-analysis of randomized controlled trials
Source: Front Endocrinol (Lausanne). 2026 Mar 26;17:1811595. doi: 10.3389/fendo.2026.1811595 (PMC13061723; doi:10.3389/fendo.2026.1811595)
Supplement: Supplementary file 4 [file Table1.docx]

**Supplementary table 1 .** Baseline characteristics of included studies

| Author year | Country | Blinding | Sample Size | | Gender  (Female/Male) | | Average Age | | | DF  Classification | | Interventions | | intervention time (Week) | Healing Rate | | Healing time  （Day） | |
| --- | --- | --- | --- | --- | --- | --- | --- | --- | --- | --- | --- | --- | --- | --- | --- | --- | --- | --- |
|  |  |  | IG | CG | IG | CG | IG | | CG | IG | CG | IG | CG |  | IG | GG | IG | GG |
| Snyder 2018 | Multicentre | Double | 172 | 164 | 35/137 | | | 59.9 | 56.4 | University of Texas grade 1A or 2A | | B | A | 12 | 22.7% | 18.3% | NA | NA |
| Driver 2017 | Multicentre | Double | 61 | 61 | 16/45 | | | 59 | 59 | University of Texas Class IA | | D | A | 12 | 56% | 49% | NA | NA |
| Armstrong 2023 | USA | Single | 50 | 50 | 14/36 | | | 60 | 57 | Wagner grade 1 | | C | A | 12 | 70% | 34% | NA | NA |
| Niederauer 2018 | USA | Double | 74 | 72 | 15/59 | 18/54 | | 56 | 56 | University of Texas Class 1A | | D | A | 12 | 32.4% | 16.7% | NA | NA |
| RG 2019 | USA & Europe | Double | 36 | 37 | 2/31 | 5/32 | | 63 | 63 | University of Texas grade 1-2 | | D | A | 12 | 42% | 14% | NA | NA |
| Ennis 2005 | USA & Canada | Double | 27 | 28 | 8/47 | | | 19-89 | | Wagner grade 1-2 | | E | A | 12 | 40.7% | 14.3% | 63.8±4.1 | 82.2±1.5 |
| Snyder 2024 | Multicenter | Single | 59 | 60 | 14/45 | 11/60 | | 58 | 56 | Wagner Grade 1-2 | | F | A | 12 | 41% | 15% | NA | NA |
| Wainstein 2011 | Israel | Double | 32 | 29 | 15/17 | 10/19 | | 62 | 62 | Wagner Grade 2–4 | | D | A | 12 | 41% | 33% | NA | NA |
| Gould 2022 | USA | Single | 50 | 50 | 23/27 | 17/33 | | 62 | 62 | Wagner Grade 1-2 | | C | H | 12 | 74% | 38% | ​​54 ±7 | 64±8 |
| Armstrong 2000 | USA | Double | 52 | 45 | 14/38 | 11/34 | | 49 | 52 | University of Texas grade 2-3  infected foot wounds | | I | A | 12 | 75% | 51% | NA | NA |
| Shetty 2022 | India | Single | 26 | 26 | 15/11 | 12/14 | | 57 | 59 | Wagner Grade 1-2 | | C | A | 12 | 84.6% | 53.8% | NA | NA |
| Maranna 2021 | India | NO | 22 | 23 | 6/16 | 6/17 | | 50 | 49 | Wagner Grade 1-2 | | G | A | 2 | 90.9% | 26.1% | 14.82 ± 7.30 | 44.57 ± 9.29 |
| Lantis 2023 | USA | Single | 51 | 51 | 15/36 | 17/34 | | 58 | 63 | University of Texas grade 1A/1C | | N | A | 12 | 56.9% | 31.4% | 50.2 ± 20.3 | 51.2±21.4 |
| Jesús 2022 | Mexico | Single | 40 | 40 | 18/22 | 21/19 | | 66 | 63 | Saint Elian grades I-III | | C | A | 4 | NA | NA | 16.1±14 | 42.7±21 |
| Nayak 2024 | India | NO | 36 | 36 | 12/24 | 13/23 | | 52 | 56 | Wagner Grade 2-3 | | K | A | 12 | 57.6% | 12.9% | NA | NA |
| Lullove 2021 | USA | Single | 24 | 25 | NA | NA | | 57 | 60 | Superficial ulcers (UT grade 1A/1C) | | C | H | 12 | 67% | 32% | NA | NA |
| Das 2024 | India | NO | 36 | 36 | 14/22 | 16/20 | | 56 | 55 | Wagner grade 1-2 | | F | A | 12 | 77.78% | 44.44% | 78.19 ± 19.11 | 96.46 ± 11.62 |
| Eraydin 2017 | Turkey | NO | 30 | 30 | 15/15 | 8/22 | | 61 | 66 | Wagner grade 1-2 | | K | A | 12 | 20% | 3% | NA | NA |
| Esmael 2023 | Egypt | NO | 15 | 15 | 9/6 | 7/8 | | 48 | 48 | Wagner grade 1-2 | | O | A | 8 | NA | NA | 47.46±12.59 | 59.18±2.71 |
| Serena 2021 | USA | NO | 81 | 64 | 26/54 | 11/53 | | 64 | 63 | IDSA 1-2 or Wagner 1-2 | | D | A | 12 | 44.4% | 28.1% | NA | NA |
| Alvarez 2006 | USA | NO | 25 | 24 | 14/11 | 13/11 | | 59 | 59 | Plantar ulcers with adequate circulation | | J | A | 12 | 72% | 38% | NA | NA |
| He 2021 | China | NO | 40 | 40/40 | 17/23 | 16/24  15/25 | | 64 | 63/62 | Wagner grade 2-3 | | L | D/H | 8 | 72.5% | 47.5%/ 42.5% | 48.39 ± 13.32 | 75.52± 25.62/76.42 ± 32.78 |
| Lantis 2021 | USA | NO | 103 | 104 | 24/79 | 20/84 | | 58 | 59 | Wagner grade 2-3 | | N | A | 12 | 45.6% | 27.9% | NA | NA |
| Qin 2019 | China | NO | 30 | 30 | 16/14 | 17/13 | | 67 | 66 | Wagner grade 2-4 | | M | H | 12 | 93.2% | 21.3% | NA | NA |

**Notes:**Country= Multicentre:≥3；Blinding: single = single-blind、Double= double-blind、NO= Unblinded；IG:Intervention group、CG:Control group；Interventions:A=Standard care or Standard Care + Placebo、B=Standard Care + Focused Extracorporeal Shock Wave Therapy、C= Standard Care +Allogeneic Skin Grafts、D= Standard Care + Gas Therapy、E= Standard Care + Ultrasound Therapy、F= Standard Care + Autologous Blood-Derived Products、G= Standard Care + Negative Pressure Therapy、H= Standard Care + Dressing Therapy、I= Standard Care + Pneumatic Therapy 、J=Standard Care + Non-contact Normal Temperature Wound Therapy、K= Standard Care + Exercise Therapy、L= Standard Care + Gas Therapy + Dressing Therapy、M= Standard Care + Autologous Blood-Derived Products + Dressing Therapy、N=Standard Care + Xenogeneic Skin Grafts、O= Standard Care + Phototherapy；NA=No date
