# Supplementary material for: Effects of non-pharmacological interventions on ulcer healing in patients with diabetic foot: a network meta-analysis of randomized controlled trials
Source: Front Endocrinol (Lausanne). 2026 Mar 26;17:1811595. doi: 10.3389/fendo.2026.1811595 (PMC13061723; doi:10.3389/fendo.2026.1811595)
Supplement: Supplementary file 5 [file Table2.docx]

Supplementary Table 2 Definition of intervention measures

| **Code** | **Name** | **The definition of specific intervention measures in this study** | **Core mechanism** |
| --- | --- | --- | --- |
| A=1 | Standard Care（SC） or Standard Care + Placebo | It includes 8 basic measures : 1 debridement ( sharp / mechanical removal of necrotic tissue ) ; 2 basic dressing ( saline gauze, etc. ) ; 3 wound cleaning and protection ; 4 routine foot protection ( loose shoes and socks, no special decompression device ) ; 5 Blood glucose control ; 6 Infection control ; 7 basic nutritional support ( non-experimental preparation ) ; 8 Basic health education. | Eliminate wound pathogenic factors, control blood glucose and infection, optimize wound bed conditions, and lay a foundation for ulcer healing |
| B=2 | Standard Care + Focused Extracorporeal Shock Wave Therapy (ESWT) | On the basis of standard nursing, combined with focused extracorporeal shock wave therapy to promote wound healing. | Stimulate local angiogenesis and tissue regeneration, improve wound blood perfusion, and alleviate tissue ischemia |
| C=3 | Standard Care + Allograft Skin（AS） | On the basis of standard nursing, the wounds were covered with allogeneic skin grafts. | Provide physical protection for the wound bed, reduce external stimulation, act as a scaffold for epithelial cell migration and proliferation, and promote wound re-epithelialization |
| D=4 | Standard Care + Gas Therapy(GT) | On the basis of standard nursing, combined with gas intervention ( such as hyperbaric oxygen, local oxygen therapy, etc. ) to improve the oxygen environment of the wound. | Optimize local wound oxygen supply, correct tissue hypoxia, inhibit anaerobic bacterial reproduction, enhance oxidative metabolism of wound cells, and promote granulation tissue growth |
| E=5 | Standard Care + Ultrasound Therapy(UT) | On the basis of standard nursing, ultrasonic therapy was used to assist wound repair. | Produce mechanical and cavitation effects, promote local microcirculation, accelerate absorption of inflammatory exudate, and enhance fibroblast activity and collagen synthesis |
| F=6 | Standard Care + Autologous Blood-Derived Products(ABDP) | On the basis of standard nursing, autologous blood-derived products ( such as platelet-rich plasma, etc. ) were used to promote healing. | Release endogenous growth factors (e.g., platelet-derived growth factor), activate wound repair signaling pathways, and promote proliferation and differentiation of fibroblasts, endothelial cells, and epithelial cells |
| G=7 | Standard Care + Negative Pressure Wound Therapy (NPWT) | On the basis of standard nursing, continuous negative pressure drainage was used to remove exudate, reduce edema and promote granulation growth. | Remove wound exudate and necrotic tissue, reduce local edema, improve wound perfusion, stimulate granulation tissue formation, and maintain a moist wound environment |
| H=8 | Standard Care + Dressing Therapy(DT) | On the basis of standard nursing, dressings with special functions ( non-basic dressings ), such as foam and alginate, were used to regulate the wound microenvironment. | Maintain a moist wound microenvironment, absorb excess exudate, isolate external bacteria, reduce wound pain, and create favorable conditions for cell proliferation and migration |
| I=9 | Standard Care + Pneumatic Therapy(PT) | On the basis of standard nursing, the blood circulation of the lower limbs was improved by a pneumatic device. | Through intermittent pneumatic compression, promote venous and lymphatic return of the lower limbs, reduce edema, improve systemic blood supply to the foot, and alleviate tissue ischemia |
| J=10 | Standard Care + Non-contact Normal-temperature Wound Therapy(NNWT) | On the basis of standard nursing, non-contact normal temperature technology was used to protect the wound. | Form a physical protective barrier on the wound surface, reduce wound dehydration and mechanical damage, maintain stability of the wound microenvironment, and avoid secondary injury |
| K=11 | Standard Care + Exercise Therapy(ET) | On the basis of standard nursing, patients were guided to carry out targeted foot exercise and improve local circulation. | Enhance muscle activity of the lower limbs and feet, promote local blood circulation, increase tissue nutrient and oxygen supply, improve wound metabolic capacity, and reduce ulcer recurrence risk |
| L=12 | Standard Care + Gas Therapy + Dressing Therapy | On the basis of standard nursing, combined with gas therapy and characteristic dressing, can synergistically improve wound healing conditions. | Synergistically optimize local oxygen supply and moist microenvironment, inhibit bacterial infection, reduce wound exudation, and jointly promote proliferation and repair of wound tissue cells |
| M=13 | Standard Care + Autologous Blood-Derived Products + Dressing Therapy | On the basis of standard nursing, autologous blood derivative products and characteristic dressings were combined to enhance the repair effect. | Sustained release of endogenous growth factors through the dressing carrier, maintain a stable moist wound environment, prolong the biological activity of growth factors, and jointly activate the sequential wound repair process |
| N=14 | Standard Care + Xenogeneic Skin Grafts（XSG） | On the basis of standard nursing, heterogeneous allogeneic skin grafts ( such as fish skin ) were used to cover the wound. | Form a biological protective barrier, protect the wound bed, absorb exudate, provide a scaffold for host repair cell adhesion and proliferation, and regulate local inflammatory response |
| O=15 | Standard Care + Phototherapy (P) | On the basis of standard nursing, specific wavelength light ( such as infrared, laser, etc. ) was used to promote wound healing. | Utilize photothermal and photochemical effects of specific wavelengths, promote local angiogenesis, reduce inflammatory response, accelerate epithelial tissue regeneration, and enhance collagen remodeling |

**Reference**：Schaper NC, van Netten JJ, Apelqvist J, et al. Practical guidelines on the prevention and management of diabetes-related foot disease (IWGDF 2023 update). Diabetes Metab Res Rev. 2024;40(3):e3657. doi:10.1002/dmrr.3657
