# Supplementary material for: Effects of non-pharmacological interventions on ulcer healing in patients with diabetic foot: a network meta-analysis of randomized controlled trials
Source: Front Endocrinol (Lausanne). 2026 Mar 26;17:1811595. doi: 10.3389/fendo.2026.1811595 (PMC13061723; doi:10.3389/fendo.2026.1811595)
Supplement: Supplementary file 6 [file Table3.docx]

**Supplementary Table 3** Results of network meta-analysis

（a）12-week healing rate


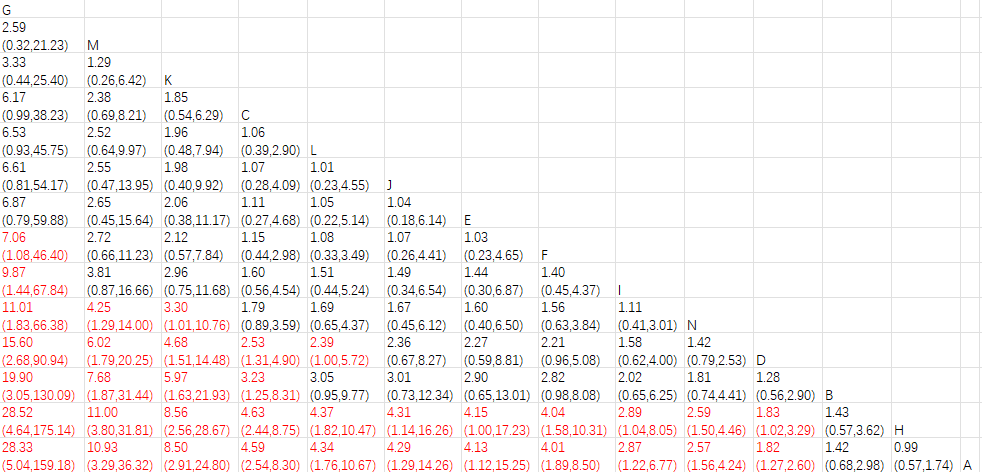


（b）healing time


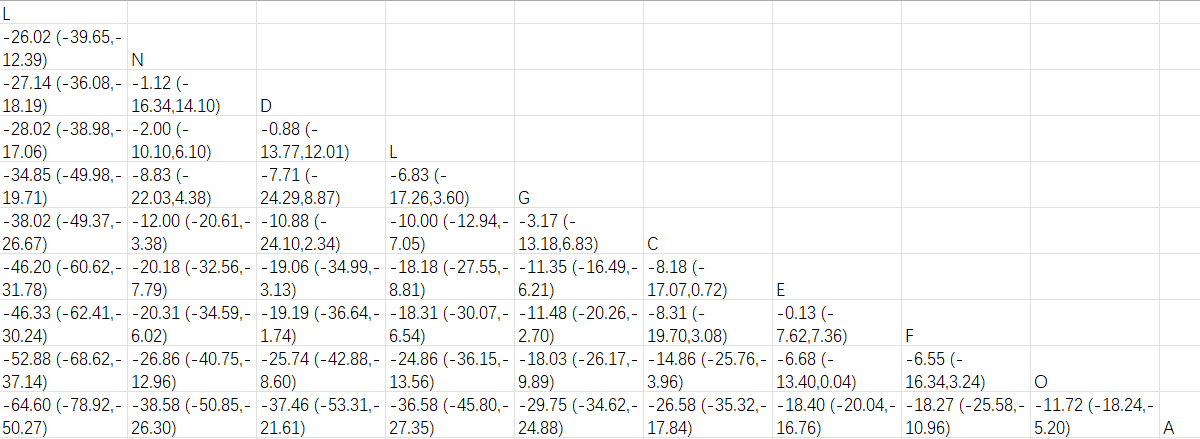


**Note**: A = Standard Care or Standard Care + Placebo; B = Standard Care + Focused Extracorporeal Shock Wave Therapy; C = Standard Care + Allograft Skin; D = Standard Care + Gas Therapy; E = Standard Care + Ultrasound Therapy; F = Standard Care + Autologous Blood-Derived Products; G = Standard Care + Negative Pressure Wound Therapy; H = Standard Care + Dressing Therapy; I = Standard Care + Pneumatic Therapy; J = Standard Care + Non-contact Normal-temperature Wound Therapy; K = Standard Care + Exercise Therapy; L = Standard Care + Gas Therapy + Dressing Therapy; M = Standard Care + Autologous Blood-Derived Products + Dressing Therapy; N = Standard Care + Xenogeneic Skin Grafts; O = Standard Care + Phototherapy.
