# Supplementary material for: Effects of non-pharmacological interventions on ulcer healing in patients with diabetic foot: a network meta-analysis of randomized controlled trials
Source: Front Endocrinol (Lausanne). 2026 Mar 26;17:1811595. doi: 10.3389/fendo.2026.1811595 (PMC13061723; doi:10.3389/fendo.2026.1811595)
Supplement: Supplementary file 7 [file Table4.docx]

Supplementary Table 4 ：GRADE Evidence for Intervention Efficacy

| **Intervention group vs Control group** | **Outcome measure** | **Study design** | **Number of studies/Total sample size** | **Effect size**  **(OR/MD, 95%CI)** | **Downgrading judgments** | **Overall certainty**  **(GRADE)** |
| --- | --- | --- | --- | --- | --- | --- |
| SC+NPWT vs SC | 12-week healing rate | RCT | 1/45  (IG=22,CG=23) | OR=28.33, 95%CI: 5.04~159.18 | 1. Imprecision: Extremely wide confidence interval (5.04~159.18) and small sample size (45 cases), 1 level downgraded; 2. Risk of bias: Blinding not implemented in the study with concerns about bias, 1 level downgraded;   3. Indirectness: Only one study available, 1 level downgraded | Very low |
| SC+ABDP+DT vs SC | 12-week healing rate | RCT | 1/60  (IG=30, CG=30) | OR=10.93, 95%CI: 3.29~36.32 | 1. Imprecision: Small sample size (60 cases) and relatively wide confidence interval, 1 level downgraded;  2. Risk of bias: No specific description of allocation concealment and blinding in the study, 1 level downgraded;  3. Indirectness: Only one study available, 1 level downgraded | Very low |
| SC+GT+DT vs SC | Healing time | RCT | 1/80  (IG=40,CG=40) | MD=-64.60, 95%CI: -78.92~-50.27 | 1. Risk of bias: Blinding not implemented in the study, 1 level downgraded; 2. Imprecision: Small sample size (40 cases in each group), 1 level downgraded;   3. Indirectness: Only one study available, 1 level downgraded | Very low |
| SC+XSG vs SC | Healing time | RCT | 2/309  (IG=154,CG=155) | MD=-38.58, 95%CI: -50.85~-26.30 | 1. Risk of bias: Blinding not implemented in the study | Moderate |

**Note：**① SUCRA ranking of the top 2 interventions by outcome measure (overall study): (1) For 12-week healing rate: SC+NPWT vs SC (SUCRA=96.1%), SC+ABDP+DT vs SC (SUCRA=86.5%); (2) For healing time: SC+GT+DT vs SC (SUCRA=100%), SC+XSG vs SC (SUCRA=79%).

② Abbreviations: IG=Intervention Group, CG=Control Group, SC=Standard Care, NPWT=Negative Pressure Wound Therapy, ABDP=Autologous Blood-Derived Products, DT=Dressing Therapy, GT=Gas Therapy, XSG=Xenogeneic Skin Grafts, OR=Odds Ratio, MD=Mean Difference, CI=Confidence Interval, RCT=Randomized Controlled Trial.

③ GRADE certainty levels: Very low (extremely low certainty), Moderate (moderate certainty).
